# Association between cytomegalovirus infection, reduced gray matter volume, and resting-state functional hypoconnectivity in major depressive disorder: a replication and extension

**DOI:** 10.1038/s41398-021-01558-6

**Published:** 2021-09-07

**Authors:** Haixia Zheng, Bart N. Ford, Rayus Kuplicki, Kaiping Burrows, Peter W. Hunt, Jerzy Bodurka, T. Kent Teague, Michael R. Irwin, Robert H. Yolken, Martin P. Paulus, Jonathan Savitz

**Affiliations:** 1 Laureate Institute for Brain Research, Tulsa, OK USA; 2 Oklahoma State Univerisity, Department of Pharmacology and Physiology, Tulsa, OK USA; 3 Department of Medicine, the University of California, San Francisco, School of Medicine, San Francisco, CA USA; 4 Stephenson School of Biomedical Engineering, University of Oklahoma, Norman, OK USA; 5 Department of Surgery, University of Oklahoma School of Community Medicine, Tulsa, OK USA; 6 Department of Psychiatry, University of Oklahoma School of Community Medicine, Tulsa, OK USA; 7 Department of Biochemistry and Microbiology, Oklahoma State University Center for Health Sciences, Tulsa, OK USA; 8 Cousins Center for Psychoneuroimmunology at UCLA, Los Angeles, CA USA; 9 Semel Institute for Neuroscience at UCLA, Los Angeles, CA USA; 10 David Geffen School of Medicine, Los Angeles, CA USA; 11 Stanley Division of Developmental Neurovirology, Johns Hopkins School of Medicine, Baltimore, MD USA; 12 Oxley College of Health Sciences, The University of Tulsa, Tulsa, OK USA

**Keywords:** Molecular neuroscience, Depression

## Abstract

Human cytomegalovirus (HCMV) is a neurotropic herpes virus known to cause neuropathology in patients with impaired immunity. Previously, we reported a reduction in the gray matter volume (GMV) of several brain regions in two independent samples of participants who were seropositive for HCMV (HCMV+) compared to matched participants who were seronegative for HCMV (HCMV−). In addition to an independent replication of the GMV findings, this study aimed to examine whether HCMV+ was associated with differences in resting-state functional connectivity (rsfMRI-FC). After balancing on 11 clinical/demographic variables using inverse probability of treatment weighting (IPTW), GMV and rsfMRI-FC were obtained from 99 participants with major depressive disorder (MDD) who were classified into 42 HCMV+ and 57 HCMV− individuals. Relative to the HCMV− group, the HCMV+ group showed a significant reduction of GMV in nine cortical regions. Volume reduction in the right lateral orbitofrontal cortex (standardized beta coefficient (SBC) = −0.32, [95%CI, −0.62 to −0.02]) and the left pars orbitalis (SBC = −0.34, [95%CI, −0.63 to −0.05]) in the HCMV+ group was also observed in the previous study. Regardless of the parcellation method or analytical approach, relative to the HCMV− group, the HCMV+ group showed hypoconnectivity between the hubs of the sensorimotor network (bilateral postcentral gyrus) and the hubs of the salience network (bilateral insula) with effect sizes ranging from SBC = −0.57 to −0.99. These findings support the hypothesis that a positive HCMV serostatus is associated with altered connectivity of regions that are important for stress and affective processing and further supports a possible etiological role of HCMV in depression.

## Introduction

Human cytomegalovirus (HCMV) is a common neurotropic herpes virus that infects up to 75% of the US population [1]. HCMV has been identified as a major cause of neuropathology in immunologically-naïve or immune-compromised patients (congenital infection, AIDS patients, transplant recipients) [2–4]. Although HCMV can evade the host immune system to establish life-long latent infections and periodically reactivate in hosts subjected to stress or with weakened immunity [5–7], HCMV-induced neuropathology has generally not been studied in medically healthy populations. However, HCMV infection has been associated with cognitive decline [8], increased risk of neurological and psychiatric disorders (i.e., stroke, depression, anxiety, schizophrenia, and bipolar disorder) [9–12], and has been hypothesized to contribute to the progression of Alzheimer’s disease [13, 14]. Furthermore, HCMV DNA was found in the brains of a percentage of immunocompetent individuals (13.9%, 5 out of 36 cases) with a variety of neuropathological changes (mostly cerebrovascular alterations) [4]. These strands of evidence raise the possibility that under certain circumstances HCMV-induced neuropathology may also occur in individuals not classically considered to be immunocompromised. Thus, it is important to investigate whether HCMV infection is playing a mechanistic role in the CNS alterations characteristic of neuropsychiatric disorders.

Individuals with major depressive disorder (MDD) may be particularly vulnerable to the reactivation of HCMV given the link between depression, stress, and impaired viral immunity [15–17]. We recently reported a reduction in the gray matter volume (GMV) of several brain regions in two independent samples of participants who were seropositive for HCMV (HCMV+) compared to matched participants who were seronegative for HCMV (HCMV−) [18]. Although only two of these regions, the left fusiform gyrus, and the right supramarginal gyrus, were replicated across both samples, the effect sizes suggested that the association between HCMV and reduced GMV might be widespread across other cortical regions such as orbitofrontal, temporal, and parietal areas. The samples in question were composed of individuals with MDD and healthy controls (HCs), but post hoc analyses showed that the MDD participants drove the association between HCMV and brain volume. In a second paper [19], we reported reduced white matter integrity in two independent HCMV + MDD samples in the left and right inferior fronto-occipital fasciculus, a large bundle of white matter fibers that connects the parietal and occipital lobe to the orbitofrontal cortex via the insula and the temporal lobe [20, 21]. It is noteworthy that the orbitofrontal cortex, insula, and temporal regions have long been documented as a target for herpes simplex encephalitis [22–24]. Together this evidence raises two important questions. First, are HCMV-associated structural brain changes also anatomically localized in orbitofrontal and temporal areas, and second, is HCMV serostatus associated with changes in brain function in MDD?

There is a growing consensus that the human brain is organized into complex networks that fundamentally support a wide range of cognitive and affective functioning [25–28], such as attention [29], memory [30], interoceptive/exteroceptive sensation [31, 32], and emotion regulation [33]. Resting-state functional connectivity (FC), that is, the low-frequency blood oxygen level-dependent (BOLD) signal temporal correlations between spatially distant brain regions, provides a straightforward approach to study the functional organization and connectivity properties of the human brain. These slow, synchronized oscillations between areas are robust [34] and collectively form complex functional networks [25–28]. For instance, the well-known default mode network reflects self-referential thinking [35]; the salience network is involved in processing emotion or monitoring for interoceptive/exteroceptive salient events [36]; the dorsal attention network and frontoparietal network (also known as the central executive network) supports top-down cognitive control [37]; and the sensorimotor network underlies somatosensory processing [38, 39]. A meta-analysis of 27 seed-based voxel-wise resting-state FC datasets yielded evidence for abnormalities in FC across and within large-scale networks in MDD, reporting hypoconnectivity within and between the salience, dorsal attention, and frontoparietal networks, but hyperconnectivity within default mode network and between the default mode network and the frontoparietal network [40]. Although the precise function of large-scale networks remains a matter of debate, resting-state FC offers a noninvasive and efficient way to explore a potentially important neurobiological signature of HCMV infection in MDD.

Here, we tested the relationship between HCMV serostatus and GMV and resting state FC in a fully independent sample composed of individuals with MDD. Our aims were threefold. First, to determine if we could replicate the association between HCMV infection and reduced GMV in the context of MDD. Second, to determine the anatomical specificity of any such HCMV effect. Third, to link anatomical findings to function using the resting-state FC analyses to corroborate and elaborate the putative impact of HCMV on the brain. Findings from the current study would provide further evidence for the hypothesis that HCMV infection is responsible for some of the neuroimaging abnormalities observed in MDD. These putative neuropathological effects could theoretically be preventable given the existence of well-tolerated anti-HCMV medications and the ongoing development of HCMV vaccines [41, 42].

## Methods

### Participants

The current study included 99 participants aged 18–55 years who received a DSM-V diagnosis of MDD (with or without a comorbid anxiety disorder) based on the Mini International Neuropsychiatric Inventory (MINI) [43]. Participants completed the Patient-Reported Outcomes Measurement Information System (PROMIS) [44] scales for depression and anxiety, Patient Health Questionnaire 9 (PHQ-9) [45] for depressive symptoms, Customary Drinking and Drug use Record (CDDR) [46] structured interview for lifetime alcohol use, as well as the childhood trauma questionnaire (CTQ) [47] for early life stress. Data were collected between October 2018 and March 2020. Exclusion criteria included: inclusion in previous GMV paper [18], MDD in full remission, comorbid psychiatric disorders (except for anxiety disorders), substance use disorders (except for alcohol use disorder), neurological disorders, unstable medical disorders, a history of moderate-to-severe traumatic brain injury at the time of data collection, a positive urine drug screen, a body mass index (BMI) <17 or >40 kg/m^2^, a history of autoimmune disorders (except hypothyroidism), missing neuroimaging data (structural and functional images) and general MRI exclusion criteria. Approval for the current study was obtained from the Western Institutional Review Board, and written informed consent was obtained from all participants.

### Anti-CMV IgG antibodies and C-reactive protein

Serum was isolated from morning blood samples following standard laboratory procedures and frozen at −80 ^o^C. Thawed samples were tested blind to diagnosis for IgG antibodies using a semiquantitative solid-phase ELISA (IBL America, catalog #EI2570-9601G). A sample was considered HCMV seropositive if it had an optical density (OD) value of 20% over the supplied cutoff standard, equivalent to approximately ten international antibody units. Due to the non-normal distribution, the OD values were quantified as plate-adjusted *z*-scores with a mean value for each plate of two and a standard deviation of one.

The concentration of C-reactive protein (CRP) was measured using whole venous blood with the Diazyme high sensitivity CRP point of care (POC) test kit (#DZ135B-SMA-discontinued) on the SMART 700 analyzer (Diazyme Laboratories). The measurement range was from 0.5 to 23 mg/L.

### Image acquisition

T1-weighted anatomical images were acquired on two identical 3 T scanners (GE Discovery MR750) using an MPRAGE sequence with the following parameters: FOV = 240 mm, 186 slices, slice thickness = 0.9 mm, voxel dimensions = 0.938 × 0.938 × 0.9 mm^3^, image matrix = 256 × 256, TR/TE = 5/2.012 ms, acceleration factor R = 2 in the phase encoding direction, flip angle = 8°.

Resting-state functional image data were acquired on the same scanners with the following parameters: Single-shot gradient-recalled echoplanar imaging (EPI) sequence with Sensitivity Encoding depicting BOLD contrast. FOV = 240 mm, slice thickness = 2.9 mm, gap = 0 mm, matrix = 128 × 128, axial slices = 39, voxel size = 1.875 × 1.875 × 2.9 mm^3^, TR/TE = 2000/27 ms, number of TRs = 180. Data were collected with two 6-min duration runs. Therefore, the total number of TRs = 180 × 2. During the acquisition, participants were instructed to “focus on the cross, clear your mind, and try not to think of anything in particular with eyes open”.

### Individual-level image processing

For T1-weighted anatomical images, cortical reconstruction and volumetric segmentation were performed using FreeSurfer version 6.0.0. [48] Whole-brain GMVs were estimated from individual anatomical images, including 68 cortical regions (34 regions per hemisphere) using the Desikan-Killiany atlas [49, 50]. Visual inspection of all cortical segmentation was performed before analysis for quality assurance purposes. FreeSurfer has been validated against histological measurements and demonstrates good test-retest reliability [51].

For resting-state functional images, ﻿ preprocessing and individual level analysis was performed using the SPM12 software (Welcome Department of Imaging Neuroscience, Institute of Neurology, London, UK) with the CONN-toolbox [52] version 19 (https://web.conn-toolbox.org) in Matlab 2016a. ﻿The standard preprocessing pipeline implemented in the CONN-toolbox was applied. The initial five scans were removed to eliminate equilibration effects. Functional images were co-registered to the structural image and normalized to Montreal Neurological Institute (MNI) space with 2 mm isotropic voxels. Slice-timing correction and head motion correction were performed. Images were smoothed with an 8 mm full width at half maximum Gaussian kernel. Band-pass filtering was set to a frequency window from 0.01 to 0.1 Hz. Potential outlier scans were identified with framewise displacement above 0.9 mm or global BOLD signal changes above 5 SD [53]. A component-based noise-correction “aCompCor” strategy [54] implemented in this toolbox was used to control physiological, and movement confounds. This denoising method applied ordinary least squares regression to regress out the noise components from the BOLD signal, including noise components from cerebral white matter and cerebrospinal areas, head motion parameters derived from preprocessing, identified outlier scans, and scan sections. One participant who had head movement estimates above 3 mm was excluded.

To relate the FC results with the GMV findings, we used the same Desikan-Killiany atlas to define the cortical regions of interest (ROI, total 68 ROIs) to compute the whole-brain ROI-to-ROI FC maps for each individual. The BOLD signal was first averaged over each defined ROI, and the *Z-*score of the correlation coefficient between each pair of ROIs was defined as FC. However, the Desikan-Killiany atlas may not be sufficient to fully capture the brain’s FC as it parcellates the brain into relatively large regions based on anatomical structure. To overcome this limitation, large-scale network-based parcellation was also used in the current study. Specifically, five commonly reported large-scale networks’ ten hub regions were used as seeds to compute the ROI-to-ROI inter-network FC (between the ten hub regions) and the seed-to-voxel whole-brain FC of large-scale networks for each individual. These network hub regions were predefined by the CONN toolbox [52] based on independent component analyses of the Human Connectome Project (497 subjects), including (1) medial prefrontal cortex and posterior cingulate cortex for the default mode network; (2) left and right lateral sensorimotor cortex for the sensorimotor network; (3) left and right anterior insula for the salience network; (4) left and right intraparietal sulcus for the dorsal attention network; and (5) left and right dorsolateral prefrontal cortex for the frontoparietal network (also known as the central executive network). Information and coordinates are summarized in Supplementary Table S1.

### Group-level statistical analysis

For the GMV replication analysis, the statistical analysis in the current study followed the identical statistical procedural used in the previous study [18]. Briefly, (1) inverse probability of treatment weighting (IPTW) [55, 56] methodology was used to balance the following independent variables to mitigate potential confounding bias: age, sex, BMI, education, income, PROMIS depression score, PROMIS anxiety score, medication status (yes/no), early-life stress (CTQ score), number of depressive episodes (MINI interview), and lifetime alcohol use (CDDR interview). The stabilized weights were estimated using the “ipw” package. (2) Standardized mean differences were calculated to examine covariate balance before and after IPTW. (3) To confirm the effect of HCMV on GMV while accounting for the weights and estimating robust standard error, weighted generalized linear regression models from the “Survey” package were used at each of the regions. The total intracranial volume (TIV) was added into the model as a covariate to adjust for individual differences in overall brain size. A total number of four missing data points for these eleven variables were imputed using the *k* nearest neighbor algorithm with *k* = 10 (R, DMwR package). The statistical significance was determined by a threshold of *p*_uncorrected_ < 0.05 (two-tailed). These statistical analyses were performed using RStudio V1.1.463 and R version 3.5.3.

For the FC group-level analyses, differences in each pair of ROI-to-ROI and Seed-to-Voxel connectivity between HCMV+ and HCMV− were tested separately using general linear models implemented in the CONN-toolbox with age, sex, and BMI as covariates. An ROI/Seed level threshold of *P*_uncorrected_ < 0.001 and a false discovery rate (FDR) multi-test correction of *P*_FDR_ < 0.05 was used as the statistical threshold to identify the significant FC alterations. Subject level ROI-to-ROI and Seed-to-Voxel connectivity data were exported to R and the effect size was estimated while adjusting for eleven measured covariates using the weighted generalized linear regression models (weights obtained from IPTW). All the significant findings without IPTW adjustment were also reported as a sensitivity analysis.

In exploratory analyses, the relationships between the significant GMV/FC findings and semiquantitative anti-HCMV IgG level and log-transformed CRP concentration were examined within the MDD HCMV+ subgroup. Additionally, the correlation coefficient between the significant GMV/FC findings and depressive symptom severity (measured by each of the nine PHQ-9 items and the total PHQ-9 score) was tested in all participants. The FDR was used for control for multiple testing.

Finally, two sensitivity analyses were carried out to test the robustness of the findings. First, general linear regression models controlling for age, sex, BMI, and TIV were performed to assess the sensitivity of observed results to the selection of balancing covariates (IPTW model). Second, we calculated E-values [57] to evaluate the robustness of the results to potential unmeasured confounding for both significant GMV and FC findings using the “EValue” package. The E-value estimates the minimum effect an unmeasured confounder would need to have to be able to explain away an observed association with the outcome of interest.

## Results

### Study population and covariates balance

Demographic and clinical characteristics before and after applying IPTW are summarized in Table 1. After applying IPTW, the demographic differences between HCMV+ and HCMV− diminished substantially (i.e., standardized mean differences between HCMV+ and HCMV− groups for eleven measured potential confounders were all less than 0.1, indicating well-balanced groups). The plot of weights and propensity score distributions in Supplementary Fig. 1 demonstrated that no extreme weights were present, and the propensity weighting achieved the balance between the HCMV+ and HCMV− groups. Although there was a lower percentage of depression without anxiety comorbidity in the HCMV+ MDD group relative to the HCMV− MDD group, the overall anxiety severity did not differ across groups (Table 1). There were no statistically significant group differences in any of the measured covariates between HCMV+ and HCMV− subgroups. There was no significant difference in medication type and depressive symptoms between the HCMV+ and HCMV− groups before and after IPTW (Table 1).

**Table 1:** Demographic and clinical characteristics of study participants before and after applying inverse probability of treatment weighting (IPTW).

	Before applying IPTW				After applying IPTW			
	HCMV−	HCMV+	*p*^a^	SMD^b^	HCMV−	HCMV+	*p*^a^	SMD^b^
*n*	57	42			57.18	41.58		
Age (mean (SD))	30.5 (11.4)	31.6 (10.8)	0.62	0.10	29.7 (11.1)	29.6 (10.1)	0.96	0.01
Sex = Male (%)	11 (19.3)	9 (21.4)	0.99	0.05	12.1 (21.1)	8.6 (20.6)	0.95	0.01
BMI (mean (SD))	25.49 (4.82)	28.72 (5.81)	0.00	0.61	26.74 (5.32)	26.63 (5.88)	0.93	0.02
Education (mean (SD))^c^	6.77 (1.55)	6.21 (1.69)	0.09	0.34	6.44 (1.68)	6.36 (1.52)	0.83	0.05
Income (mean (SD))^d^	10.51 (1.39)	10.69 (0.66)	0.44	0.17	10.60 (1.15)	10.70 (0.64)	0.54	0.10
Depress severity (mean (SD))^e^	63.50 (6.80)	62.29 (6.99)	0.39	0.18	63.26 (7.09)	62.89 (6.83)	0.81	0.05
Anxiety severity (mean (SD))^f^	62.79 (6.46)	63.13 (5.71)	0.79	0.06	62.75 (6.35)	62.96 (5.74)	0.87	0.04
Anxiety comorbidity (%)^g^			0.03	0.77			0.05	0.75
Dep	23 (40.4)	6 (14.3)			21.1 (36.9)	5.7 (13.8)		
Dep + Alc	0 (0.0)	1 (2.4)			0.0 (0.0)	1.0 (2.4)		
Dep + Anx	24 (42.1)	17 (40.5)			25.2 (44.1)	16.4 (39.3)		
Dep + Panic	1 (1.8)	1 (2.4)			1.2 (2.1)	0.8 (1.9)		
Dep + PTSD	4 (7.0)	9 (21.4)			5.7 (10.0)	8.5 (20.4)		
Dep + Soc phobia	5 (8.8)	8 (19.0)			4.0 (6.9)	9.2 (22.2)		
Medicated (%)^h^	21 (36.8)	10 (23.8)	0.25	0.29	17.0 (29.8)	11.9 (28.6)	0.91	0.03
Medication type (%)^g^								
NDRI	5 (8.8)	1 (2.4)			4.1 (7.1)	1.3 (3.2)		
SNRI	3 (5.3)	1 (2.4)			2.5 (4.4)	0.8 (2.0)		
SSRI	12 (21.1)	5 (11.9)			9.7 (17.0)	7.3 (17.5)		
other	1 (1.8)	3 (7.1)			0.7 (1.3)	2.5 (5.9)		
CTQ (mean (SD))^i^	45.1 (15.1)	50.8 (19.7)	0.11	0.33	48.5 (17.2)	47.6 (18.8)	0.83	0.05
Number of episodes (mean (SD))^j^	4.14 (3.49)	4.41 (3.49)	0.70	0.08	4.14 (3.55)	4.19 (3.51)	0.95	0.01
Alcohol use (mean (SD))^k^	4.43 (2.32)	4.14 (2.37)	0.55	0.12	4.14 (2.43)	4.16 (2.21)	0.97	0.01
HCMV IgG level (mean (SD))^l^	1.25 (0.23)	2.96 (0.59)	<0.01	3.80	1.28 (0.23)	2.93 (0.52)	<0.01	4.11
Log CRP (mean (SD))^m^	0.72 (1.05)	0.69 (0.96)	0.86	0.04	0.81 (1.06)	0.47 (0.91)	0.11	0.35
Depressive symptoms^n^								
Anhedonia	1.40 (0.80)	1.17 (0.62)	0.11	0.33	1.36 (0.77)	1.17 (0.59)	0.17	0.27
Depressed mood	1.35 (0.92)	1.43 (0.63)	0.64	0.10	1.29 (0.92)	1.39 (0.59)	0.52	0.13
Sleep problems	1.89 (0.99)	1.60 (1.11)	0.16	0.29	1.93 (0.96)	1.68 (1.14)	0.30	0.24
Tiredness	2.14 (0.90)	1.83 (0.88)	0.09	0.35	2.10 (0.87)	1.94 (0.87)	0.40	0.18
Changes in appetite	1.02 (0.88)	1.21 (1.02)	0.31	0.21	1.08 (0.85)	1.28 (1.03)	0.34	0.21
Feelings of inadequacy	1.42 (0.89)	1.48 (0.77)	0.75	0.07	1.39 (0.85)	1.54 (0.76)	0.38	0.19
Concentration problems	1.30 (0.87)	1.21 (0.98)	0.65	0.09	1.27 (0.88)	1.33 (0.96)	0.80	0.06
Psychomotor changes	0.46 (0.68)	0.31 (0.60)	0.27	0.23	0.47 (0.69)	0.35 (0.63)	0.41	0.18
Suicidality	0.44 (0.76)	0.31 (0.52)	0.34	0.20	0.45 (0.79)	0.31 (0.50)	0.35	0.20
PHQ-9 total score	11.42 (4.47)	10.55 (4.03)	0.32	0.21	11.33 (4.54)	10.98 (3.89)	0.71	0.08

*MDD* major depressive disorder, *HCMV*− human cytomegalovirus seronegative, *HCMV*+ human cytomegalovirus seropositive, *SMD* standardized mean difference, *BMI* body mass index, *Dep* depression (no comorbidity), *Dep* + *Alc* depression with alcohol dependence disorder, *Dep* + *GAD* depression with generalized anxiety disorder, *Dep* + *PD* depression with panic disorder, *Dep* + *PTSD* depression with post-traumatic stress disorder, *Dep* + *Soc Phobia* depression with social phobia. *NDRI* norepinephrine-dopamine reuptake inhibitor, *SNRI* selective norepinephrine reuptake inhibitor, *SSRI* selective serotonin reuptake inhibitor, *CTQ* childhood trauma questionnaire, *CRP* C-reactive protein.

^a^Calculated using *X*^2^ test for categorical variables and two-tailed *t*-test for continuous variables.

^b^The standardized mean differences less than 0.1 reveals a negligible imbalance.

^c^Measured by ordered categories. For full categories, see Supplementary Table S4.

^d^Household income (log-transformed).

^e^PROMIS depression T score was used.

^f^PROMIS anxiety T score was used.

^g^Data obtained from the MINI clinical interview.

^h^Medicated defined as taking psychotropic medication.

^i^Childhood trauma questionnaire total score was used.

^j^Measured by MINI interview. Subjects who had over ten episodes were treated as had ten episodes.

^k^Lifetime alcohol usage was used (log-transformed). Data obtained from the CDDR interview.

^l^HCMV IgG level *z-*score was used.

^m^CRP concentration (log-transformed).

^n^Measured by patient health questionnaire.

### GMV differences between HCMV+ and HCMV−

**Fig. 1:**
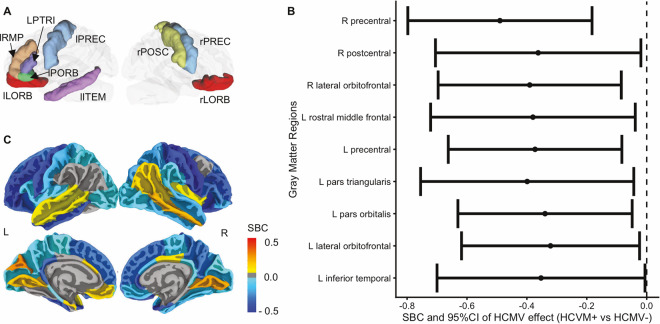
Gray matter volume differences between HCMV+ and HCMV−. **A** Illustration of regions that showed an effect of HCMV. Nine regions were significantly smaller in HCMV+ versus HCMV− subjects at *p*_uncorrected_ < 0.05. Two out of these nine regions (lPORB and rLORB) were also significantly decreased in the original study at *p*_uncorrected_ < 0.05. **B** Standardized beta coefficient (equivalent to Cohen’s d) as effect size with 95% CI is estimated from the IPTW adjusted regression model. The robust standard error was used to calculate the 95%CI. **C** Mapping of the HCMV effect at all the cortical regions without thresholding (for exact values see Supplementary Table S1). Colors represent the standardized beta coefficients estimated from the IPTW adjusted regression model. They range from −0.5 to 0.5, indicating that the mean gray matter volume of the HCMV+ subgroup in the given region increased or decreased by 0.5 standard deviations relative to the HCMV− subgroup. Blue colors represent smaller gray matter volumes in HCMV+ groups, whereas yellow-red colors represent larger gray matter volumes in HCMV+ groups. Consistent with the original findings, relative to HCMV− subjects, HCMV+ subjects showed widespread smaller gray matter volumes, most prominently in orbitofrontal, temporal, and parietal regions. rLORB right lateral orbitofrontal gyrus, rPREC right precentral gyrus, rPOSC right postcentral gyrus, lLORB right lateral orbitofrontal gyrus, lPORB left pars orbitalis gyrus, lITEM left inferior temporal gyrus, lRMP left rostral middle frontal gyrus, LPTRI left pars triangularis, lOREC left precentral gyrus.

Consistent with our previous publication, relative to HCMV− subjects, HCMV+ subjects showed widespread reductions in GMVs, most prominently in orbitofrontal, temporal, and parietal regions (Fig. 1C). Relative to HCMV− subjects, HCMV+ subjects showed a reduction of GMV in 49 out of 68 cortical regions although only nine of these regions were statistically significant (*p*_uncorrected_ < 0.05, Fig. 1A, B and Table 2), i.e., the left inferior temporal gyrus (SBC = −0.35, [95%CI, −0.70 to −0.01], note that SBC is a measure of differences between groups in units of standard deviation), left lateral orbitofrontal gyrus (SBC = −0.32, [95%CI, −0.62 to −0.02]), left pars orbitalis gyrus (SBC = −0.34, [95%CI, −0.63 to −0.05]), left pars triangularis (SBC = −0.40, [95%CI, −0.75 to −0.04]), left precentral gyrus (SBC = −0.37, [95%CI, −0.66 to −0.08]), left rostral middle frontal gyrus (SBC = −0.38, [95%CI, −0.72 to −0.04]), right lateral orbitofrontal gyrus (SBC = −0.39, [95%CI, −0.70 to −0.08]), right postcentral gyrus (SBC) = −0.36, [95%CI, −0.71 to −0.02]), and right precentral gyrus (SBC = −0.49, [95%CI, −0.80 to −0.18]). No regions were significantly larger in the HCMV+ versus the HCMV− group (Supplementary Table S2). Taken together, the significant volume differences observed were of small-to-medium effect size.

**Table 2:** HCMV effect on gray matter volume and resting-state functional connectivity.

	Association with HCMV			Correlation with IgG		Correlation with CRP	
Gray matter volume (Regions)	SBC^a^	95%CI^b^	*p*_uncorrected_	*r*	*p*_uncorrected_	*r*	*p*_uncorrected_
L inferior temporal gyrus	−0.35	−0.70 to −0.01	0.05*	−0.05	0.75	−0.09	0.59
L lateral orbitofrontal gyrus	−0.32	−0.62 to −0.02	0.04*	0.00	0.98	−0.24	0.14
L pars orbitalis	−0.34	−0.63 to −0.05	0.02*	−0.09	0.58	−0.31	0.05*
L pars triangularis	−0.40	−0.75 to −0.04	0.03*	−0.08	0.61	−0.32	0.04*
L precentral gyrus	−0.37	−0.66 to −0.08	0.01**	0.08	0.60	−0.13	0.43
L rostral middle frontal gyrus	−0.38	−0.72 to −0.04	0.03*	−0.10	0.52	−0.24	0.13
R lateral orbitofrontal gyrus	−0.39	−0.70 to −0.08	0.01**	−0.02	0.90	−0.16	0.32
R postcentral gyrus	−0.36	−0.71 to −0.02	0.04*	0.07	0.68	0.07	0.67
R precentral gyrus	−0.49	−0.80 to −0.18	0.002**	0.08	0.63	−0.13	0.44
**Functional connectivity (ROI-to-ROI)**	**SBC**	**95%CI**	***p***_**FDR**_	***r***	***p***_**uncorrected**_	***r***	***p***_**uncorrected**_
L Insula–R Postcentral gyrus	−0.73	−1.11 to −0.35	0.005***	−0.02	0.90	−0.18	0.26
R Insula–L Postcentral gyrus	−0.65	−1.06 to −0.24	0.04***	−0.19	0.23	−0.12	0.47
R Insula – R Postcentral gyrus	−0.57	−1.00 to −0.13	0.04***	0.05	0.76	−0.07	0.65
L Postcentral gyrus– R Superior temporal gyrus	−0.68	−1.05 to −0.31	0.04***	−0.22	0.17	−0.20	0.22
R Postcentral gyrus – R Inferior parietal gyrus	0.69	0.28–1.10	0.04***	−0.19	0.23	−0.35	0.03*
**(Network Seed-to-Voxel)**							
Sensorimotor_.R Lateral sensorimotor cortex_ – L Frontal operculum Cluster	−0.99	−1.36 to −0.62	<0.001***	0.14	0.36	0.30	0.06
Salience._R Anterior insula_ – L Postcentral Cluster	−0.91	−1.28 to −0.54	0.003***	−0.03	0.85	−0.26	0.10

*ROI* region of interest, *R* right, *L* left.

^*^P_uncorrected_ < 0.05. ^**^P_uncorrected_ < 0.01. ^***^P_FDR_ < 0.05

^a^Standardized beta coefficient, equivalent to Cohen’s *d*. SBC of 1 indicates that the mean gray matter volume of the HCMV + subgroup is 1 standard deviation different from the HCMV− subgroup. A negative value indicates HCMV + < HCMV− and a positive value indicates HCMV + > HCMV−.

^b^95%CI, 95% confidence interval, robust standard errors were used to calculate 95%CI.

### FC differences between HCMV + and HCMV−

**Fig. 2:**
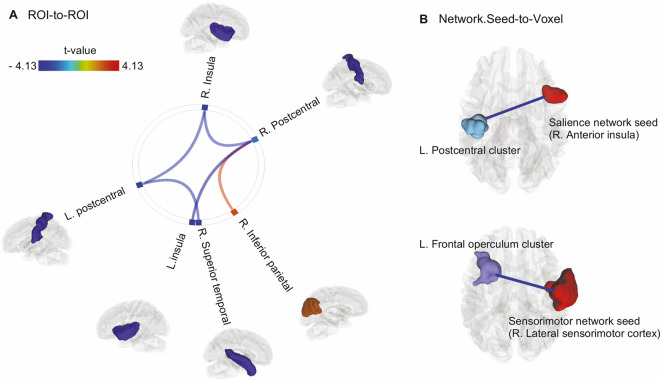
Functional connectivity differences between HCMV+ and HCMV−. **A** Illustration of anatomical structural (Desikan-Killiany atlas)-based ROI-to-ROI analyses findings (*p*_FDR_ < 0.05). The figure represents connections were increased (orange-red color) or decreased (green-blue color) connectivity was found in HCMV+ relative to HCMV− participants. **B** Functional network-based Seed-to-Voxel analyses identified two FC pairs that were significantly lower in HCMV+ relative to HCMV− participants (voxel-level *p*_uncorrected_ < 0.001, cluster level *p*_FDR_ < 0.05). That is 1. from right anterior insula for the salience network to left postcentral cluster (cluster size = 274 voxels, peak coordinate x, y, z = −36, −16, +50); 2. from right lateral sensorimotor cortex for the sensorimotor network to the left frontal operculum cluster (cluster size = 405 voxels, peak coordinate x, y, z = −36, +22, +08). The ROI/seed regions and the identified clusters were exported as binary maps and rendered using DSI-studio for visualization. Effect sizes (standardized beta coefficient) ranged from −0.57 to −0.99. Please see Table 2 for details.

Five pairs of anatomical-based ROI-to-ROI FC showed significant differences between HCMV+ and HCMV− groups (*p*_FDR_ < 0.05, Fig. 2A and Table 2). Relative to HCMV− subjects, HCMV+ subjects showed hypo-connectivity in the left insula-to- right postcentral gyrus (SBC = −0.73, [95%CI, −1.11 to −0.35]), right insula-to-left postcentral gyrus (SBC = −0.65, [95%CI, −1.06 to −0.24]), right insula-to-right postcentral gyrus (SBC = −0.57, [95%CI, −1.00 to −0.13]), and left postcentral gyrus-to-right superior temporal gyrus (SBC = −0.68, [95%CI, −1.05 to −0.31]). Relative to HCMV− subjects, HCMV+ subjects also showed hyper-connectivity in the right postcentral gyrus-to-right inferior parietal gyrus (SBC = 0.69, [95%CI, 0.28 to 1.10]). Thus, in comparison to the volumetric changes, ROI-to-ROI FC differences were of medium effect size.

Large-scale network-based Seed-to-Voxel analyses revealed that HCMV+ subjects showed significant hypo-connectivity within the salience network and the sensorimotor network relative to HCMV− subjects (voxel-level *p*_uncorrected_ < 0.001, cluster level *p*_FDR_ < 0.05, Fig. 2B and Table 2). The strength of FC between the right anterior insula of the salience network and the left postcentral cluster was also significantly lower in the HCMV+ group relative to the HCMV− group (cluster size = 274 voxels, SBC = −0.91, [95%CI, −1.28 to −0.54]). Further, the strength of FC between the right sensorimotor cortex and the left frontal operculum cluster was significantly lower in the HCMV+ group relative to the HCMV− group (cluster size = 405 voxels, SBC = −0.99, [95%CI, −1.36 to −0.62]). No significant within-network differences were found between HCMV+ and HCMV− when seeds were placed in the hub regions of default mode network, central executive network, and dorsal attention network. No significant network differences (between the ten predefined network hub regions) were found between HCMV+ and HCMV− using a threshold of *p*_FDR_ < 0.05. Results significant at *p*_uncorrected_ < 0.05 are reported in Supplementary Fig. S2 to show the putative inter-network connection alterations in association with HCMV serostatus. There was hypoconnectivity between sensorimotor network, salience network, and dorsal attention network, and hyperconnectivity between default mode network and frontoparietal network observed when using a threshold of *p*_uncorrected_ < 0.05 (Supplementary Fig. S2). The effect sizes of these connectivity differences were large.

### Correlations between HCMV IgG level, CRP, and neuroimaging findings

Correlation analyses were performed in the HCMV+ subgroup for the nine cortical regions of reduced GMV, the five pairs of ROI-to-ROI FC, and the two pairs of Seed-to-Voxel FC that showed significant alterations. Although no significant correlations survived FDR correction, CRP concentration was inversely correlated with the left pars orbitalis volume (*r* = −0.31, *p*_uncorrected_ = 0.049) and the left pars triangularis volume (*r* = −0.32, *p*_uncorrected_ = 0.044), as well as the FC between right postcentral gyrus and right inferior parietal gyrus (*r* = −0.35, *p*_uncorrected_ = 0.03). There were no significant correlations between semiquantitative HCMV IgG level and any neuroimaging findings (Table 2).

### Correlations between specific depressive symptoms and neuroimaging findings

Exploratory correlation analyses between specific depressive symptoms and neuroimaging findings were performed in all the MDD subjects. Only the regions that showed significant GMV/FC difference between HCMV+ and HCMV− groups were tested. There were no significant correlations between specific depressive symptoms (indexed by each of PHQ-9 items and the total PHQ-9 score) after FDR correction. The correlation coefficient values are summarized in Supplementary Fig. S3.

### Sensitivity analysis

Sensitivity analysis using a general linear regression model controlling for age, sex, BMI, and TIV yielded similar results with a larger effect size than the IPTW model (Supplementary Table S3). Based on our dataset and the observed effect size, the estimated E-value ranged from 2.02 to 4.01, suggesting that the observed HCMV effects were at least moderately robust to potential unmeasured confounders (Supplementary Table S3). The E-value methodology estimates the joint minimum strength of association on the risk ratio scale that an unmeasured confounder must have with both treatment and outcome in order to fully explain away an observed effect. Thus, to explain away the observed effect of HCMV on GMV and FC, a putative unmeasured confounder would need to increase the likelihood of having a smaller GMV/ lower FC and being HCMV+ by at least 2.02 to 4.01 times each.

## Discussion

This study aimed to examine whether HCMV+ was associated with differences in GMV or with altered resting state FC and yielded three main findings. (1) Consistent with our previous report of GMV reductions in HCMV+ MDD participants, nine regions were significantly reduced in volume in HCMV+ compared with HCMV− participants matched on up to 11 different potential confounding variables. No regions were significantly larger in HCMV+ versus HCMV− participants. (2) Of these regions, the reduction in the volume of the right lateral orbitofrontal cortex and the left pars orbitalis (the orbital component of inferior frontal gyrus) was observed in the HCMV+ group in this study was also present in our previous publication [18]. (3) the HCMV+ group showed hypoconnectivity between the hubs of the sensorimotor network (bilateral postcentral gyrus) and the hubs of the salience network (bilateral insula). Taken together, these findings support the hypothesis that positive HCMV serostatus is associated with altered connectivity of regions that are important for stress and affective processing and further supports a possible etiological role of HCMV in depression.

Reduced GMV in the orbitofrontal cortex appeared to be most robustly associated with HCMV infection in the context of MDD. The orbitofrontal cortex receives and integrates sensory-visceromotor inputs [58, 59], and has been demonstrated to be involved in decision making, reward prediction, emotion identification, and the hedonic experience [60–64]. Indeed, reduced GMV or thickness of the orbitofrontal cortex has been reported in the ENIGMA consortium MDD cohort as well as meta-analyses of MDD populations [65–67]. Although not statistically significant in our previous study, we also found that the middle frontal gyrus, sensorimotor cortex, and inferior temporal gyrus showed significant reductions in GMV in the HCMV+ group relative to the HCMV− group. These results are consistent with our previous finding that HCMV associated GMV reductions are distributed across cortical regions but are most prominent in orbitofrontal, temporal, and parietal regions. Decreased GMV or thickness in these regions was also observed in other neurotropic viral infections, such as herpes simplex virus, measles virus, and HIV [68–71], raising a possibility that these regions maybe particularly vulnerable to viral infection. The apparent similarity in the anatomical distribution of HCMV-associated structural brain changes and herpes simplex encephalitis-associated neuropathology [22–24] needs further testing in postmortem brain samples.

We used both anatomical-based parcellation (ROI-to-ROI analyses) and network-based parcellation (Seed-to-Voxel analyses) to investigate alterations of FC in the context of HCMV infection. Both approaches highlighted hypoconnectivity between the salience network and sensorimotor network in the HCMV+ group versus the HCMV− group. The insula (particularly, the anterior insula) and the salience network have long been recognized for their essential role in interoceptive/exteroceptive salient stimuli detection, emotion regulation, and coordination of other brain large-scale network dynamics to guide behavior (i.e., dynamic switching between default mode network and central executive network) [36, 37, 72]. In the FC literature, hypoconnectivity in the salience network or insula cortex has been consistently observed in MDD [36, 73] relative to HCs. The primary somatosensory cortex, the postcentral gyrus, has direct control over the stress response as it has a direct neuronal connection to the adrenal medulla [74]. Recent studies have reported decreased FC between the insula and postcentral gyrus in depressed patients with bipolar disorder relative to HCs [75, 76]. Further, the circulating IL-6 concentration was negatively correlated with the FC between the insula and postcentral gyrus [76]. Additionally, the inter-network connectivity (FC between the ten predefined hub regions) also revealed a hypoconnectivity between the salience network and the sensorimotor network, hypoconnectivity between the sensorimotor network and the dorsal attention network, and hyperconnectivity between the default mode network and the frontoparietal network in the HCMV+ group relative to the HCMV− group (Supplementary Fig. S2). Although consistent with previous findings from FC meta-analyses in MDD [40], these results did not survive FDR correction and therefore need to be treated with caution. Together, our finding raises the possibility that HCMV infection may impair the functional coupling between the salience and sensorimotor network systems, which are thought to play a vital role in stress response and emotional processing.

Leboyer and colleagues previously reported an inverse correlation between HCMV IgG titer and right hippocampal volumes in patients with schizophrenia and bipolar disorder [11]. Here, we found inverse correlations between CRP concentration and pars orbitalis volume and the pars triangularis volume, as well as the FC between the right postcentral gyrus and right inferior parietal gyrus. However, we did not observe a significant association between HCMV IgG level and any of the neuroimaging findings. This may be because our measure of IgG antibody titer is only a semiquantitative, indirect measure of HCMV shedding. Further, IgG antibodies have a half-life of <30 days. Thus, the signal-to-noise ratio of these correlation analyses are likely to be low. Specific markers of a viral infection such as CXCL10/IP-10, macrophage activation such as sCD14, or HCMV encoded microRNAs may be better markers of HCMV activity than IgG level or CRP concentration, however, more research is needed to answer this question [77, 78]. We did not observe any significant association between depressive symptom severity measured by PHQ-9 items and the structural/functional changes in HCMV+ subjects. Although the structural and functional alterations discussed above were consistently found in patients with MDD, the precise neurocircuitry compromised in MDD has not been identified. A recent conceptual framework has proposed that the orbitofrontal cortex, insula, and sensorimotor cortex are at the core of integrating sensory-visceromotor inputs and encoding subjective emotion and cognitive state [64, 79–81]. However, the items of the PHQ-9 (and other clinical scales) do not map well onto neurocircuitry.

The cellular mechanisms underlying the association between HCMV seropositivity and brain alterations in MDD are unclear. There are at least five possible interpretations: First, periodic reactivation of HCMV in the brain may directly damage neural tissue as HCMV can infect endothelial cells of the blood–brain barrier as well as glia, neurons, and neural precursor cells [82–85]. Second, periodic reactivation of HCMV in the brain or the periphery may contribute to an inflammatory process that in turn leads to a reduction in GMV [86]. Third, HCMV infection may trigger autoimmunity (e.g., via molecular mimicry) and lead to tissue pathology [87, 88]. Fourth, HCMV is capable of manipulating host immunity and modifying immune function which could in theory lead to tissue damage. For instance, HCMV encodes a unique IL-10 homolog, which is 27% identical to human IL-10 and can bind to IL-10 receptors and profoundly impact host immune signaling and T-cell response [89–91]. Fifth, HCMV may be a harmless bystander associated with another unknown factor that is the actual cause of the observed structural and functional deficits. Future nonhuman primate or experimental human studies are warranted to investigate the underlying mechanisms through which HCMV infection putatively leads to GMV and connectivity reduction.

This study has several limitations. First, HCMV seropositivity is an indirect measure of viral activity. Future studies with more specific markers of CMV reactivation (e.g., HCMV encoded microRNAs [78]) are needed to better evaluate the association between HCMV activity and brain structural/functional alterations. Second, we cannot differentiate between the acute and cumulative effects of HCMV on GMV and FC. Third, potential unmeasured confounders such as other viral infections or socioeconomic disadvantage that co-occur with HCMV may account for observed associations. Nevertheless, sensitivity analysis against unmeasured confounders using E-value methodology suggests that the HCMV effect observed in the current study is at least moderately robust against unmeasured confounders.

In sum, after balancing up to 11 potential confounding factors, we replicate the finding that HCMV infection is associated with GMV reduction in the context of MDD and provide the first evidence that HCMV infection is associated with hypoconnectivity between regions involved in stress response and emotional processing. The results remain robust in several sensitivity analyses. While a causal conclusion cannot be drawn given the cross-sectional design, our findings provide further support to the hypothesis that HCMV infection may play an etiological role in a vulnerable subgroup of MDD patients. Future studies with larger longitudinal samples or clinical trials with anti-HCMV treatments are warranted to validate the findings and explore clinical applications.

## Supplementary information

Supplemental material

## Data Availability

The full neuroimaging processing script and statistical analyses code are available from the corresponding author on reasonable request.
