# Supplementary material for: Association between cytomegalovirus infection, reduced gray matter volume, and resting-state functional hypoconnectivity in major depressive disorder: a replication and extension
Source: Transl Psychiatry. 2021 Sep 7;11:464. doi: 10.1038/s41398-021-01558-6 (PMC8423754; doi:10.1038/s41398-021-01558-6)
Supplement: Supplementary file 1 — Supplemental material [file 41398_2021_1558_MOESM1_ESM.docx]

**Supplementary Contents**

Table of Contents

[Supplementary Table S1. Large-scale network seeds information. 2](#_Toc64316750)

[Supplementary Table S2. Effect of HCMV serostatus on Gray Matter Volume 3](#_Toc64316751)

[Supplementary Table S3. Sensitivity analyses results summary 5](#_Toc64316752)

[Supplementary Table S3. Education measurement 6](#_Toc64316753)

[Supplementary Figure S1. IPTW diagnostic plots. 7](#_Toc64316754)

[Supplementary Figure S2. Association between HCMV infection and inter-network functional connectivity strength. 8](#_Toc64316755)

[Supplementary Figure S3. The correlation coefficient between gray matter volume, functional connectivity, and the depressive symptoms 9](#_Toc64316756)

### Supplementary Table S1. Large-scale network seeds information.

| Networks | Seeds | MNI coordinates | | |
| --- | --- | --- | --- | --- |
|  |  | x | y | z |
| Default mode network | Medial prefrontal cortex | +1 | +55 | -3 |
|  | Posterior cingulate cortex | +1 | -61 | +38 |
| Sensorimotor network | Lateral sensorimotor cortex(R) | -55 | -12 | +29 |
|  | Lateral sensorimotor cortex (L) | +56 | -10 | +29 |
| Salience network | Anterior insula (L) | -44 | +13 | +1 |
|  | Anterior insula (R) | +47 | +14 | 0 |
| Dorsal attention network | Intraparietal sulcus (L) | -39 | -43 | +52 |
|  | Intraparietal sulcus (R) | +39 | -42 | +54 |
| Frontoparietal network | Lateral prefrontal cortex (L) | -43 | +33 | +28 |
| (Central executive network) | Lateral prefrontal cortex (R) | +41 | +38 | +30 |

Abbreviation: L, left. R, right. MNI, Montreal Neurological Institute.

### Supplementary Table S2. Effect of HCMV serostatus on Gray Matter Volume

| **Regions** | **SBC^a^** | **Std.Error^b^** | **95% CI** | ***p^c^*** |
| --- | --- | --- | --- | --- |
| L bank of the superior temporal sulcus | 0.04 | 0.22 | -0.40 ~ 0.47 | 0.87 |
| L caudal anterior cingulate | 0.12 | 0.22 | -0.32 ~ 0.55 | 0.61 |
| L caudal middle frontal gyrus | -0.37 | 0.20 | -0.76 ~ 0.02 | 0.07 |
| L cuneus | 0.26 | 0.22 | -0.18 ~ 0.70 | 0.24 |
| L entorhinal | 0.11 | 0.19 | -0.25 ~ 0.47 | 0.56 |
| L fusiform | 0.03 | 0.18 | -0.33 ~ 0.38 | 0.88 |
| L inferior parietal lobule | 0.03 | 0.17 | -0.31 ~ 0.37 | 0.88 |
| L inferior temporal gyrus | -0.35 | 0.18 | -0.70 ~ -0.01 | 0.05 |
| L isthmus cingulate cortex | -0.31 | 0.17 | -0.63 ~ 0.02 | 0.07 |
| L lateral occipital gyrus | -0.06 | 0.17 | -0.41 ~ 0.28 | 0.72 |
| L lateral orbitofrontal | -0.32 | 0.15 | -0.62 ~ -0.02 | 0.04 |
| L lingual | 0.15 | 0.19 | -0.22 ~ 0.51 | 0.44 |
| L medial orbitofrontal | 0.11 | 0.18 | -0.25 ~ 0.47 | 0.55 |
| L middle temporal gyrus | 0.04 | 0.18 | -0.31 ~ 0.40 | 0.81 |
| L parahippocampal | -0.04 | 0.20 | -0.43 ~ 0.35 | 0.85 |
| L paracentral | -0.04 | 0.20 | -0.43 ~ 0.34 | 0.82 |
| L pars opercularis | -0.11 | 0.21 | -0.52 ~ 0.29 | 0.58 |
| L pars orbitalis | -0.34 | 0.15 | -0.63 ~ -0.05 | 0.02 |
| L pars triangularis | -0.40 | 0.18 | -0.75 ~ -0.04 | 0.03 |
| L pericalcarine | -0.09 | 0.22 | -0.51 ~ 0.33 | 0.68 |
| L postcentral | -0.17 | 0.19 | -0.55 ~ 0.21 | 0.39 |
| L posterior cingulate cortex | -0.03 | 0.21 | -0.45 ~ 0.39 | 0.89 |
| L precentral | -0.37 | 0.15 | -0.66 ~ -0.08 | 0.01 |
| L precuneus | -0.09 | 0.17 | -0.42 ~ 0.24 | 0.60 |
| L rostral anterior cingulate cortex | -0.03 | 0.21 | -0.45 ~ 0.39 | 0.87 |
| L rostral middle frontal gyrus | -0.38 | 0.17 | -0.72 ~ -0.04 | 0.03 |
| L superior frontal gyrus | -0.28 | 0.16 | -0.59 ~ 0.03 | 0.08 |
| L superior parietal lobule | -0.08 | 0.15 | -0.37 ~ 0.22 | 0.60 |
| L superior temporal gyrus | 0.06 | 0.17 | -0.28 ~ 0.40 | 0.75 |
| L supramarginal gyrus | 0.02 | 0.18 | -0.33 ~ 0.38 | 0.89 |
| L frontal pole | -0.15 | 0.18 | -0.51 ~ 0.21 | 0.40 |
| L temporal pole | -0.37 | 0.19 | -0.74 ~ 0.00 | 0.05 |
| L transverse temporal | -0.05 | 0.18 | -0.39 ~ 0.30 | 0.79 |
| L insula | -0.12 | 0.14 | -0.41 ~ 0.16 | 0.40 |
| R bank of the superior temporal sulcus | 0.10 | 0.22 | -0.32 ~ 0.52 | 0.65 |
| R caudal anterior cingulate | -0.16 | 0.20 | -0.56 ~ 0.23 | 0.42 |
| R caudal middle frontal gyrus | 0.02 | 0.21 | -0.39 ~ 0.42 | 0.94 |
| R cuneus | -0.04 | 0.19 | -0.41 ~ 0.33 | 0.84 |
| R entorhinal | -0.36 | 0.21 | -0.78 ~ 0.06 | 0.10 |
| R fusiform | -0.13 | 0.15 | -0.43 ~ 0.16 | 0.37 |
| R inferior parietal lobule | 0.11 | 0.17 | -0.22 ~ 0.43 | 0.52 |
| R inferior temporal gyrus | -0.18 | 0.17 | -0.51 ~ 0.15 | 0.29 |
| R isthmus cingulate cortex | -0.06 | 0.19 | -0.42 ~ 0.31 | 0.76 |
| R lateral occipital gyrus | -0.10 | 0.17 | -0.43 ~ 0.23 | 0.56 |
| R lateral orbitofrontal | -0.39 | 0.16 | -0.70 ~ -0.08 | 0.01 |
| R lingual | 0.20 | 0.16 | -0.12 ~ 0.53 | 0.22 |
| R medial orbitofrontal | -0.14 | 0.16 | -0.44 ~ 0.17 | 0.38 |
| R middle temporal gyrus | 0.17 | 0.18 | -0.19 ~ 0.53 | 0.35 |
| R parahippocampal | 0.01 | 0.19 | -0.37 ~ 0.39 | 0.96 |
| R paracentral | -0.11 | 0.18 | -0.46 ~ 0.23 | 0.53 |
| R pars opercularis | -0.30 | 0.22 | -0.73 ~ 0.12 | 0.16 |
| R pars orbitalis | -0.18 | 0.20 | -0.58 ~ 0.22 | 0.38 |
| R pars triangularis | -0.27 | 0.21 | -0.68 ~ 0.15 | 0.21 |
| R pericalcarine | -0.16 | 0.18 | -0.51 ~ 0.19 | 0.38 |
| R postcentral | -0.36 | 0.17 | -0.71 ~ -0.02 | 0.04 |
| R posterior cingulate cortex | 0.08 | 0.20 | -0.31 ~ 0.48 | 0.68 |
| R precentral | -0.49 | 0.16 | -0.80 ~ -0.18 | 0.00 |
| R precuneus | -0.27 | 0.16 | -0.58 ~ 0.03 | 0.08 |
| R rostral anterior cingulate cortex | -0.35 | 0.19 | -0.72 ~ 0.02 | 0.07 |
| R rostral middle frontal gyrus | -0.33 | 0.19 | -0.71 ~ 0.04 | 0.08 |
| R superior frontal gyrus | -0.29 | 0.15 | -0.59 ~ 0.01 | 0.06 |
| R superior parietal lobule | -0.20 | 0.17 | -0.53 ~ 0.13 | 0.23 |
| R superior temporal gyrus | -0.18 | 0.17 | -0.50 ~ 0.15 | 0.29 |
| R supramarginal gyrus | -0.09 | 0.16 | -0.41 ~ 0.23 | 0.57 |
| R frontal pole | -0.30 | 0.19 | -0.67 ~ 0.07 | 0.11 |
| R temporal pole | -0.45 | 0.23 | -0.91 ~ 0.00 | 0.05 |
| R transverse temporal | -0.11 | 0.17 | -0.45 ~ 0.22 | 0.50 |
| R insula | 0.03 | 0.18 | -0.32 ~ 0.38 | 0.86 |

Abbreviation: L, left; R, right; ^a^ Standardized beta coefficient. SBC of 1 indicates that the mean gray matter volume of the HCMV+ subgroup is 1 standard deviation different from the HCMV- subgroup. A negative value indicates HCMV+ < HCMV-, and a positive value indicates HCMV+ > HCMV-. ^b^ Robust standard error; ^c^ unadjusted *p*.

###

### Supplementary Table S3. Sensitivity analyses results summary

|  | **Effect of HCMV without**  **IPTW adjustment**  **(Estimated from GLM)^a^** | | | **Sensitivity to Unmeasured confounder** | |
| --- | --- | --- | --- | --- | --- |
| **Gray Matter Volume**  **(Regions)** | **SBC^b^** | **95%CI^c^** | ***p*_uncorrected_** | | **E-value** |
| L inferior temporal gyrus | -0.40 | -0.74 ~ -0.06 | 0.02* | | 2.10 |
| L lateral orbitofrontal gyrus | -0.32 | -0.57 ~ -0.08 | 0.01** | | 2.02 |
| L pars orbitalis | -0.31 | -0.62 ~ -0.01 | 0.05* | | 2.07 |
| L pars triangularis | -0.40 | -0.74 ~ -0.06 | 0.02* | | 2.23 |
| L precentral gyrus | -0.37 | -0.62 ~ -0.12 | 0.004** | | 2.16 |
| L rostral middle frontal gyrus | -0.35 | -0.65 ~ -0.04 | 0.03* | | 2.18 |
| R lateral orbitofrontal gyrus | -0.45 | -0.72 ~ -0.18 | 0.001** | | 2.21 |
| R postcentral gyrus | -0.35 | -0.67 ~ -0.02 | 0.04* | | 2.13 |
| R precentral gyrus | -0.46 | -0.73 ~ -0.18 | 0.002** | | 2.50 |
| **Functional Connectivity**  **(ROI-to-ROI)** | **SBC** | **95%CI** | ***p*_FDR_** | | **E-value** |
| L Insula – R Postcentral gyrus | -0.82 | -1.21 ~ -0.44 | 0.005*** | | 3.29 |
| R Insula – L Postcentral gyrus | -0.69 | -1.09 ~ -0.29 | 0.04*** | | 3.02 |
| R Insula – R Postcentral gyrus | -0.66 | -1.06 ~ -0.26 | 0.04*** | | 2.74 |
| L Postcentral gyrus– R Superior temporal gyrus | -0.68 | -1.07 ~ -0.28 | 0.04*** | | 3.12 |
| R Postcentral gyrus – R Inferior parietal gyrus | 0.66 | 0.27 ~ 1.06 | 0.04*** | | 3.15 |
| **(Network.Seed-to-Voxel)** |  |  |  | |  |
| Sensorimotor.R Lateral sensorimotor cortex – L Frontal operculum Cluster | -0.93 | -1.30 ~ -0.56 | <0.001*** | | 4.35 |
| Salience.R Anterior insula – L Postcentral Cluster | -0.98 | -1.35 ~ -0.61 | 0.003*** | | 4.01 |

Abbreviation: ROI, region of interest; R, right; L, left; ^a^GLM, general linear regression model, controlling for age, sex, and BMI. Additionally, for gray matter volumes, the total intracranial volume was added as covariates. ^b^ Standardized beta coefficient, equivalent to Cohen’s d. SBC of 1 indicates that the mean gray matter volume of the HCMV+ subgroup is 1 standard deviation different from the HCMV- subgroup. A negative value indicates HCMV+ < HCMV-, and a positive value indicates HCMV+ > HCMV-. ^c^ 95%CI, 95% confidence interval. * *P*_uncorrected_ less than 0.05. ** *P*_uncorrected_ less than 0.01. *** *P*_FDR_ < 0.05.

### Supplementary Table S3. Education measurement

Education was measured by 11 categories and treated as a continuous variable:

1. No school through kindergarten
2. Grade 1-11
3. Grade 12, no diploma
4. Regular HS diploma
5. GED or equivalent
6. Some college, no degree
7. Associate's degree
8. Bachelor's degree
9. Master's degree
10. Professional degree beyond a bachelor's
11. Doctoral degree

### Supplementary Figure S1. IPTW diagnostic plots.

### Supplementary Figure S2. Association between HCMV infection and inter-network functional connectivity strength.

The figure illustrated the association between HCMV infection and inter-network functional connectivity strength. Three pairs of inter-network connectivity were identified by using the threshold of *p*_uncorrected_ < 0.05. Relative to HCMV- subjects, HCMV+ subjects showed a hypoconnectivity between the salience network (right anterior insula as seed) and the sensorimotor network (left lateral sensorimotor cortex as seed, t = -0.24, *p*_uncorrected_ = 0.02), hypoconnectivity between the sensorimotor network (left lateral sensorimotor cortex as seed) and the dorsal attention network (left intraparietal sulcus as seed, t = -2.2, *p*_uncorrected_ = 0.03). Relative to HCMV- subjects, HCMV+ subjects also showed a hyperconnectivity between the default mode network (medial prefrontal cortex as seed) and the frontoparietal network (left dorsolateral prefrontal cortex as seed, t = 2.53, *p*_uncorrected_ = 0.01).

### Supplementary Figure S3. The correlation coefficient between gray matter volume, functional connectivity, and the depressive symptoms


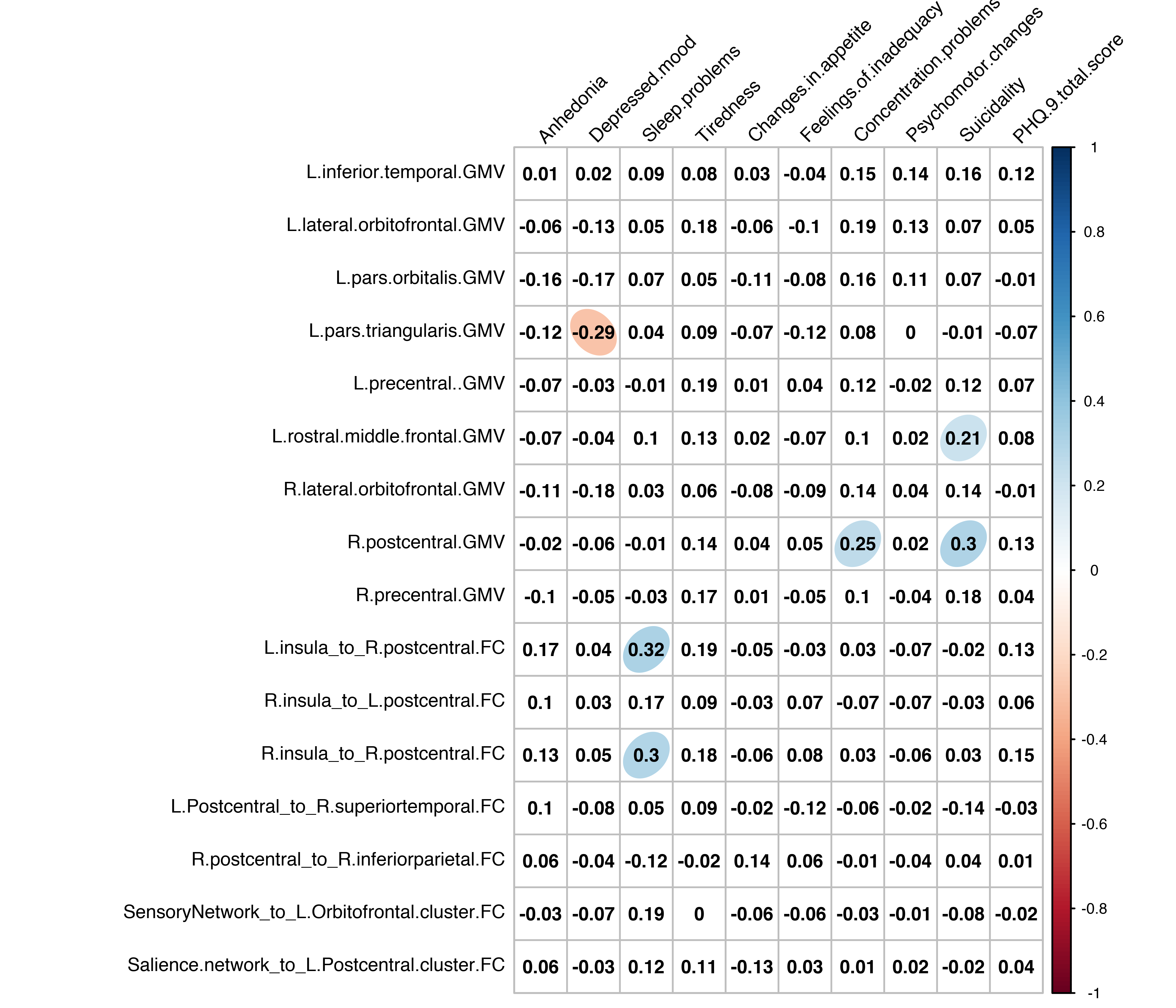


Figure summarized the correlation coefficient (Pearson’s r value) between the cortical gray matter volume, resting-state functional connectivity, and the depressive symptoms (measured by each of the nine PHQ-9 items and the total PHQ-9 score). Correlation analyses were performed in all the MDD subjects. Only the regions that showed significant difference between HCMV seropositive and HCMV seronegative groups were tested. Color circled area indicates that the r value passed the threshold of *p*_uncorrected_ < 0.05. The blue color indicates a positive association, while the orange to red color indicates a negative association. Abbreviation: L, left; R, right; GMV, gray matter volume; FC, functional connectivity.
